# Presence of *Burkholderia pseudomallei* in the ‘Granary of Myanmar’

**DOI:** 10.3390/tropicalmed4010008

**Published:** 2019-01-04

**Authors:** Tun Tun Win, Khine Khine Su, Aye Min Than, Zaw Min Htut, Khin Phyu Pyar, Elizabeth A. Ashley, David A. B. Dance, Kyaw Myo Tun

**Affiliations:** 1 Department of Preventive and Social Medicine, Defence Services Medical Academy, Mingalardon 11021, Myanmar; myo.kyaw.tun@gmail.com; 2 Department of Microbiology, Defence Services Medical Academy, Mingalardon 11021, Myanmar; drkhinekhinesu@gmail.com (K.K.S.); ayeminthant124@gmail.com (A.M.T.); 3 Department of Microbiology, Military Institute of Nursing and Paramedical Sciences, Mingalardon 11021, Myanmar; arlulay0345@gmail.com; 4 Department of Medicine, Defence Services Medical Academy, Mingalardon 11021, Myanmar; khinphyupyar@gmail.com; 5 Myanmar Oxford Clinical Research Unit, Yangon 11201, Myanmar; liz@tropmedres.ac; 6 Centre for Tropical Medicine and Global Health, Nuffield Department of Clinical Medicine, Old Road, Campus, University of Oxford, Oxford OX3 7FZ, UK; david.d@tropmedres.ac; 7 Lao-Oxford-Mahosot Hospital-Wellcome Trust Research Unit, Microbiology Laboratory, Mahosot Hospital, Vientiane, Laos; 8 Faculty of Infectious and Tropical Diseases, London School of Hygiene and Tropical Medicine, London WC1E 7HT, UK

**Keywords:** melioidosis, *Burkholderia pseudomallei*, Myanmar

## Abstract

Melioidosis is a frequently fatal infectious disease caused by the Gram negative bacillus *Burkholderia pseudomallei.* Although it was originally discovered in Myanmar, the disease disappeared from sight for many decades. This study focuses on detection of *B. pseudomallei* in soil in selected sampling sites in an attempt to start to fill the gaps in the current status of our knowledge of the geographical distribution of *B. pseudomallei* in soil in Myanmar. This cross-sectional study consists of 400 soil samples from 10 selected study townships from two major paddy growing regions. Bacterial isolation was done using a simplified method for the isolation of *Burkholderia pseudomallei* from soil. In this study, only 1% (4/400) of soil samples were found to be positive; two of four were found at 90 cm depth and another two positive samples were found at 30 cm and 60 cm. This survey has confirmed the presence of environmental *B. pseudomallei* in Myanmar indicating that the conditions are in place for melioidosis acquisition.

## 1. Introduction

Melioidosis is a frequently fatal infectious disease caused by the Gram negative bacillus *Burkholderia pseudomallei* [1]. It was originally discovered by Whitmore and Krishnaswami at Rangoon (Yangon) General Hospital (RGH) in 1911 [2,3]. Historically, melioidosis was an important disease in Myanmar and Krishnaswami with more than 200 cases reported at RGH by 1917 [4]. Subsequently, the disease remained unidentified for many decades. Since 2002, some 90 years on from the first discovery of melioidosis, small numbers of cases have been reported sporadically from Yangon, Mandalay and Magway regions [5,6,7,8,9,10,11,12,13,14,15]. 

*B. pseudomallei* is an environmental saprophyte, frequently found in rice fields, with a strong association between clinical disease and presence of *B. pseudomallei* in soil [16]. The disease is highly endemic in North East Thailand and Northern Australia regions [17,18,19], but has been reported with increasing frequency from other South East Asian countries (e.g., Cambodia, Lao PDR, Malaysia, Singapore and Vietnam) where infection is usually thought to have been acquired from soil [18,20,21,22,23]. The global burden of melioidosis is believed to be greatly underestimated, and evidence for the presence of *B. pseudomallei* in the environment is incomplete: Myanmar has to date only been categorized as “probably” endemic for melioidosis as a result of a dearth of reports of environmental isolation of *B. pseudomallei* [16]. 

In Myanmar, 70% of the population reside in rural areas and rice cultivation is the main occupation of rural dwellers. Moreover, both agricultural and non-agricultural workers in rural areas have regular contact with soil and surface water without protective footwear [24]. However, despite the high risk of *B. pseudomallei* infection amongst farmers elsewhere [25,26,27], to date there has been no report of melioidosis in farmers in Myanmar. However, 78% of Myanmar migrant workers to Thailand were seropositive by the Indirect Hemagglutination Assay (IHA), suggesting that exposure to *B. pseudomallei* was common in Myanmar [28]. It was also recently reported that 3.2% of febrile patients from the delta region of Myanmar had *B. pseudomallei*-specific antibodies suggestive of active infection [29]. Furthermore, the Ministry of Health and Sports reported that the prevalence of diabetes mellitus, a strong risk factor for melioidosis, was 10.5% in Myanmar in 2014 according to the “STEP survey” [30], and the increasing prevalence of diabetes is likely to increase the risk of people in Myanmar contracting *B. pseudomallei* infection. In summary, whilst it appears that melioidosis is still likely to be endemic in Myanmar, the true epidemiology is still unknown. 

In order to define geographical areas of melioidosis risk, environmental sampling to detect the presence of *B. pseudomallei* is a fundamental step [31]. Preliminary results of a soil survey in Myanmar conducted by Win et al. in 2017–2018 suggested that *B. pseudomallei* was present in Yangon, Kayin and Mon regions [11]. In an earlier unpublished environmental study in 2016, *B. pseudomallei* was found in 42% of soil samples in agricultural farms of Thanlyin and Hmawbi townships in Yangon Region [10]. In another unpublished environmental study from five urban townships of Yangon Region, 4 of 125 soil samples were culture positive for *B. pseudomallei* [32]. This study aimed to detect the presence of environmental *B. pseudomallei* in selected sampling sites in an attempt to start to fill the gaps in our knowledge of the geographical distribution of *B. pseudomallei* in soil in Myanmar.

### Description of Study Area

The Ayeyawaddy and Bago regions are the two main regions for paddy growing among the fourteen regions of Myanmar and were therefore selected for this study. Ayeyawaddy Region, with an area of 35,031.9 square kilometers, has a population of 6.1 million and accounts for 12.0% of the country’s population and is organized into 33 townships. Bago Region, with an area of 39,404.4 square kilometers, has a population of 4.9 million and accounts for 9.5% of the country’s population. It is organized into 28 townships. The rural population is 86% in Ayeyawaddy Region and 78% in Bago Region respectively. Agriculture is the major economic activity of both regions: Ayeyawaddy is the top paddy producer in the country and Bago is the second, with the result that together they are commonly known as “the granary of Myanmar” [28]. The average temperatures in the dry season are 32.8 °C and 33.6 °C in Ayeyawaddy and Bago, respectively. In 2017, the average rainfall was 2681 mm per year countrywide [33].

## 2. Materials and Methods

Soil collection was undertaken in both rice fields and land that was classified as ‘disused’ for agriculture purposes (defined as land which had not been used for growing paddy or other crops for a minimum of 10 years). Soil sampling was performed between November 2017 and February 2018 (the dry season). A total of 400 soil samples were taken from 10 selected study townships. A convenience sampling approach was used at select sites to be sampled due to considerations of the sample transportation period, such that all laboratory procedures could be started on the day of sample collection. To maximize the clinical relevance of the findings, site selection targeted areas where human exposure was most likely to occur i.e., residential areas or rice fields. Five study townships were selected from each region based on local maps and then four study sites were chosen from each township by consultation with inhabitants regarding cultivation and use of fertilizer in fields. Fields that were intensively fertilized were avoided because it can interfere with the presence of *B. pseudomallei* in soil [19,22]. GPS coordinates of sampling sites were recorded in decimal degrees by a DNR Garmin GPS device to an accuracy of within 20 feet. Waypoints were transferred into ArcGIS 10.4.1 software and mapping was created by ArcMap.

### Soil Sampling, Culture and Bacterial Identification

The laboratory procedures of consensus guidelines for environmental sampling for *B. pseudomallei* recommended by the Detection of Environmental *Burkholderia pseudomallei* Working Party (DEBWorP) was used in this study but numbers of soil samples per field were adapted as follows [16]. At each study site, nine holes were dug by a mechanical auger to different depths; three at 30cm, three at 60cm and three at 90cm depths because of the evidence that *B. pseudomallei* may be found at different soil depths depending on the season. Samples were collected at the center of each sampling point with a collecting cup and put into a pre-labelled zip lock plastic bag. These holes were 5 m apart, using a fixed interval sampling grid. Site location and elevation were recorded. Two samples (each weighing 10 g) were taken from each hole. Thus, a total of 18 samples were taken from 9 holes at each study site. Sampling instruments were cleaned between each use by rinsing with domestic water to remove visible debris, cleaned with 70% alcohol and dried with air before next use. The sampled soil was kept at ambient temperature (24–32 °C) and away from direct sunlight as it was transported to the laboratory for same day processing. Two methods were used for detection of *B. pseudomallei:* (i). Each sample was cultured individually. (ii). Samples from nine holes were pooled together in one plastic bag and the pooled sample was cultured. Ten grams of individual soil samples were put into a screw-capped tube together with 10 mL of TBSS-C50 broth [16] and mixed with a vortex mixer for 30 s. The pooled 90 g soil samples were mixed with 90 mL of TBSS-C50 broth and vortexed as above. Broths were incubated at 40 °C for 48 h after which one 10 μL loopful of supernatant was inoculated onto an Ashdown agar plate and streaked to achieve single colonies. Plates were incubated at 40 °C for 7 days and examined every 24 h. Colonies suspected to be *B. pseudomallei* (initially pinpoint, clear and pale pink, changing to become pinkish-purple, flat, and slightly dry or with a metallic sheen) were further characterized by oxidase test and antimicrobial sensitivity tests for gentamicin, colistin and co-amoxiclav. Presumptive identification of colonies with a positive oxidase test and characteristic antibiogram (resistance to gentamicin and colistin with susceptibility to co-amoxiclav) were confirmed as *B. pseudomallei* by a highly specific latex agglutination test. Both latex positive and negative colonies with the appearance of *B. pseudomallei* were identified by API 20NE as *Burkholderia thailandensis* if positive for arabinose assimilation [34,35,36]. The study protocol was approved by the ethical review committee of Defence Services Medical Academy (Letter No. 25/Ethics 2017 issued on 15 October 2017).

## 3. Results

A total of 400 soil samples were taken from 10 selected study townships, and 4/400 (1%) soil samples were culture positive for *B. pseudomallei.* All these samples were collected in the dry season and from three different depths (30 cm, 60 cm, 90 cm). Minimum elevation (4 feet) in the Pathein township to maximum elevation (164 feet) in the Phyu township was noted. A total of 20/40 (50%) sampling sites were rice fields and the other 50% were disused land (residential areas, pastureland and a playground next to a school). The most common soil types were clay soil and silty soil (18/40 (45%) and 12/40 (30%), respectively). Eight of 40 (20%) were loamy soil type and 2 out of 40 (5%) were sandy soil type. In the Pathein township (Ayeyawaddy), two soil isolates were detected in silty soil in a residential area (elevation 51 feet). In Eainme Township (Ayeyawaddy), one soil isolate was identified in pastureland with silty soil (elevation 48 feet). The fourth positive soil sample was from a paddy field in the Bago township in Bago Region (Table 1). In addition, *B. thailandensis* was isolated from 17 out of 400 samples (4.25%) including silty, clay and loamy soil. Detailed information on soil sampling sites provided as supplementary data.

### Mapping of B. pseudomallei and B. thailandensis Distribution

**Figure 1:**
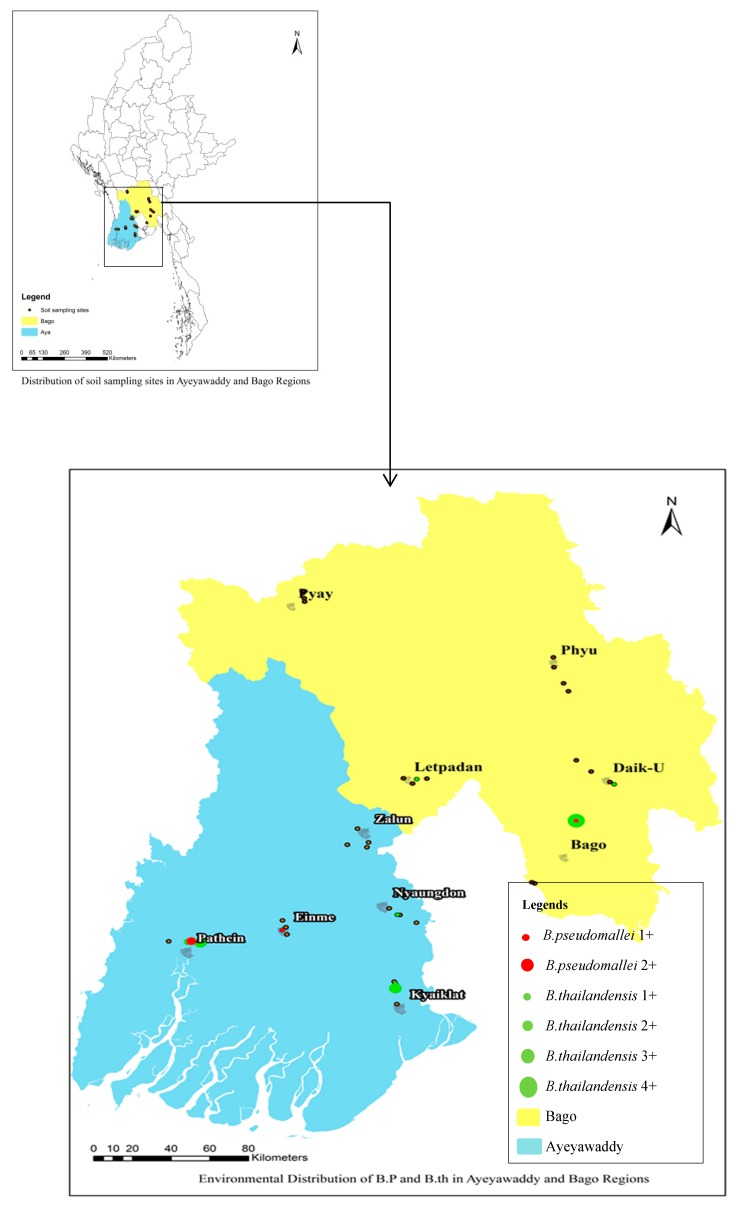
Map of *B. pseudomallei* and *B. thailandensis* from soil sampling sites in Ayeyawaddy and Bago regions.

In two townships (Pathein and Bago) both *B. pseudomallei* and *B. thailandensis* were found in the soil. These two townships are the most populated cities in both regions. In terms of study sites, co-existence of *B. pseudomallei* and *B. thailandensis* was found only in (Site No.26). Neither *B. pseudomallei* nor *B. thailandensis* were found in three study townships (Figure 1). In this figure, 1+ means only one sample was positive in the study site and 2+ means two samples were positive at this site. *B. pseudomallei* was found in a maximum of two samples (2+) in one study site and *B. thailandensis* was found in a maximum of four samples (4+) in one study site.

*B. pseudomallei* was isolated from different soil depths, both rice fields and disused land (Table 2).

## 4. Discussion

The aim of this study was to confirm the presence of *B. pseudomallei* in the soil in Myanmar and contribute to current knowledge regarding the geographical distribution of *B. pseudomallei* in soil. The results confirmed that the organism is still present in Myanmar in the rice-farming regions around Yangon and infection in humans is likely to be under-recognized, as in neighboring countries [18,20,22]. However, there are a number of limitations of this study. Although we found only a 1% (4/400) positivity rate, it is now recognized that the consensus culture method may have sub-optimal sensitivity in some areas [37]. Pooling specimens in an attempt to simplify the approach to environmental sampling was unsuccessful as we failed to detect *B. pseudomallei* from the pooled samples from all positive sites. Our study was conducted in the dry season, whereas melioidosis is more frequent during the rainy season [38,39]. However, in some studies the frequency of bacterial isolation from the environment was higher in the dry season than the rainy season [40]. In the laboratory, *B. pseudomallei* grows in soil across a pH range of 5–8. Generally, soil is near neutral pH in the rainy season and slightly acidic in the dry season [39,41], but we were unable to measure soil pH in this study. Other factors such as land use and presence of animals in the sampling area have also been associated with soil positivity rates for *B. pseudomallei* [42]. Several studies have demonstrated that soil positivity and an increased risk of human melioidosis occur in flooding-prone areas [19,21]. In recent years, heavy rain, flooding and cyclones have occurred regularly in Myanmar. The areas included in this study regularly experience flooding and cyclones, conditions which are likely to predispose to *B. pseudomallei* infection [43,44,45].

In this study, 3 of the 4 positive soil samples were collected from 2 residential areas and 1 pastureland. Domestic animals or livestock in human residential areas may contribute to the presence of the organism [19,22,40,46]. In addition, both soil-positive residential areas were shaded by trees and the positive residential area in Pathein Township contained some banana plants. The pastureland was also covered with grass and was moist and contaminated with animal excreta in some areas. This meant that these soil samples were high in moisture even in the dry season. A soil survey in Laos found that moist rich soil was associated with *B. pseudomallei* isolation [18]. However, the other positive sample was from a rice field that was less moist compared with the culture- positive disused land. Unfortunately, we cannot prove any associations in this study because of the low positivity rate. Another possible explanation for the low positivity rate in rice fields is because chemicals or fertilizers may have impacted on the survival of *B. pseudomallei* [19,22,47,48]. In this study we excluded rice fields with a history of extensive use of fertilizers or chemicals, but none of the fields were completely free of fertilizer use. Several reports have suggested that a wide range of environmental factors might influence the distribution of *B. pseudomallei* [49,50,51,52]. Only gross soil texture and altitude were measured in this study. Ayeyawaddy and Bago regions are major paddy producing areas in Myanmar and most of the soil is clay or silty. Three of 4 positives were found in silty soils and 1 positive was from clay soil, but it is impossible to draw any conclusions regarding associations between soil textures and positivity rate because of the low positivity rate. Unfortunately, we were unable to measure other physical, biological and chemical characteristics of the soil in this study. The literature on this topic is contradictory, with some studies associating the presence of *B. pseudomallei* with acrisols or with salinity [38]. The positive soil isolates were detected at a range of altitudes between 48 to 63 feet, consistent with other environmental studies [18,40].

*B. pseudomallei* was isolated from different depths in this study. Two of four (50%) positives were found at 90 cm depth and the other two positives were found at 30 cm and 60 cm respectively. Previous studies have reported that *B. pseudomallei* could be found at depths ranging from the surface to 90 cm [38], although the surface isolation rate is usually lower, perhaps related to the soil moisture or the activity of UV light [51,53,54,55]. 

*B. thailandensis* is a non-pathogenic soil-dwelling bacterium which is genetically closely related to *B. pseudomallei.* In this study *B. thailandensis* was found in 17 out of 400 soil samples. Co-existence of *B. pseudomallei* and *B. thailandensis* was uncommon at the same sampling sites. Twelve of seventeen *B. thailandensis*-positive samples were found at different study sites, but in several cases both *B. thailandensis* and *B. pseudomallei* were detected in the same site. This is consistent with findings from a Thai study [56].

## 5. Conclusions

In conclusion, this cross-sectional study has confirmed the presence of environmental *B. pseudomallei* in Myanmar indicating that the conditions are in place for melioidosis to be acquired in Myanmar. These results contribute to knowledge of the global distribution of *B. pseudomallei.* The detection of *B. pseudomallei* in environmental samples is highly method-dependent, and seasonal variation, geographical variation, physical and chemical properties of soil and farming techniques all probably influence its distribution. Further studies are needed to extend our understanding of the most important factors that impact on melioidosis risk in Myanmar. 

## Figures and Tables

**Table 1:** Details of 40 sampling sites in Ayeyawaddy and Bago regions.

Township (Site No.)	Sample (n)	Site Location	Elevation (Feet)	Land Use	Soil Texture	No. of Positive *B.p* Samples	No. of Positive *B.th* Samples
Pathein (1)	10	N 16.02851 E 95.67233	51	Residential area	Silty	2	0
(2)	10	N 16.83364 E 94.64797	77	Playground next to school	Sandy	0	0
(3)	10	N 16.83090 E 94.73723	4	Rice Field	Loam	0	2
(4)	10	N 16.82640 E 94.79514	51	Rice Field	Loam	0	3
Eainme (5)	10	N 16.91685 E 95.19268	32	Pastureland	Loam	0	0
(6)	10	N 16.95689 E 95.17681	97	Rice Field	Clay	0	0
(7)	10	N 16.89923 E 95.17532	48	Pastureland	Silty	1	0
(8)	10	N 16.87407 E 95.19691	12	Pastureland	Silty	0	0
Nyaung-don (9)	10	N 16.94337 E 95.79916	31	Residential area	Loam	0	0
(10)	10	N 16.98861 E 95.72256	21	Pastureland	Silty	0	0
(11)	10	N 16.99021 E 95.71066	36	Rice Field	Clay	0	1
(12)	10	N 17.02851 E 95.67233	16	Rice Field	Clay	0	0
Kyaiklat (13)	10	N 16.59514 E 95.69595	16	Rice Field	Loam	0	0
(14)	10	N 16.58426 E 95.70167	26	Rice Field	Clay	0	0
(15)	10	N 16.55549 E 95.70099	15	Residential area	Clay	0	0
(16)	10	N 16.46198 E 95.70800	21	Residential area	Clay	0	0
Zalun (17)	10	N 17.38901 E 95.56879	29	Rice Field	Loam	0	0
(18)	10	N 17.41841 E 95.57671	49	Rice Field	Silty	0	1
(19)	10	N 17.40408 E 95.47816	88	Playground next to school	Silty	0	3
(20)	10	N 17.49949 E 95.52522	19	Residential area	Silty	0	0
Daik-U (21)	10	N 17.77481 E 96.69761	62	Rice Field	Loam	0	0
(22)	10	N 17.76138 E 96.71742	49	Rice Field	Clay	0	1
(23)	10	N 17.83690 E 96.61082	66	Pastureland	Clay	0	0
(24)	10	N 17.90338 E 96.54168	103	Playground next to school	Clay	0	0
Bago (25)	10	N 17.55262 E 96.53234	57	Playground next to school	Silty	0	0
(26)	10	N 17.54622 E 96.54114	63	Rice Field	Clay	1	5
(27)	10	N 17.18251 E 96.33514	21	Rice Field	Clay	0	0
(28)	10	N 17.17599 E 96.34814	27	Residential area	Clay	0	0
Phyu (29)	10	N 18,31143 E 96.50533	118	Pastureland	Clay	0	0
(30)	10	N 18.35832 E 96.48242	123	Rice Field	Clay	0	0
(31)	10	N 18.45323 E 96.43761	164	Rice Field	Clay	0	0
(32)	10	N 18.51181 E 96.43520	155	Rice Field	Clay	0	0
Letpandan (33)	10	N 17.79526 E 95.84736	100	Rice Field	Silty	0	0
(34)	10	N 17.79198 E 95.80048	88	Residential area	Silty	0	1
(35)	10	N 17.76587 E 95.78111	110	Rice Field	Silty	0	0
(36)	10	N 17.79686 E 95.73823	68	Rice Field	Clay	0	0
Pyay (37)	10	N 18.84134 E 95.27945	140	Playground next to school	Clay	0	0
(38)	10	N 18.86131 E 95.27753	116	Rice Field	Loam	0	0
(39)	10	N 18.90398 E 95.26804	142	Pastureland	Silty	0	0
(40)	10	N 18.89064 E 95.27018	119	Residential area	Sandy	0	0
**(40 sites)**	**n = 400**					**4**	**17**

*B.p* = *Burkholderia pseudomallei*, *B.th* = *Burkholderia thailandensis*.

**Table 2:** Distribution of *B. pseudomallei* by land use and soil depths.

Land Use	Number of Isolated *B. pseudomallei* in Different Soil Depths			Total
	30 cm	60 cm	90 cm	
Disused Land	1	0	2	3
Rice Field	0	1	0	1
**Total**	1	1	2	4

## Supplementary Materials

Available online at http://www.mdpi.com/2414-6366/4/1/8/s1.
